# How Adding Chlorhexidine or Metallic Nanoparticles Affects the Antimicrobial Performance of Calcium Hydroxide Paste as an Intracanal Medication: An In Vitro Study

**DOI:** 10.3390/antibiotics10111352

**Published:** 2021-11-05

**Authors:** Kadiatou Sy, Kevimy Agossa, Mickaël Maton, Henry Chijcheapaza-Flores, Bernard Martel, Florence Siepmann, Etienne Deveaux, Nicolas Blanchemain, Christel Neut

**Affiliations:** 1 U1008—Controlled Drug Delivery Systems and Biomaterials, Inserm, CHU Lille, University of Lille, 59000 Lille, France; kevimy.agossa@univ-lille.fr (K.A.); mickael.maton@univ-lille.fr (M.M.); henry.chijcheapazaflores.etu@univ-lille.fr (H.C.-F.); florence.siepmann@univ-lille.fr (F.S.); etienne.deveaux@univ-lille.fr (E.D.); nicolas.blanchemain@univ-lille.fr (N.B.); 2 UMR 8207, UMET—Unité Matériaux et Transformations, CNRS—Centre National de la Recherche Scientifique, INRA—Institut National de la Recherche Agronomique, ENSCL—Ecole Nationale Supérieure de Chimie de Lille, University of Lille, 59655 Lille, France; bernard.martel@univ-lille.fr; 3 U1286 Infinite—Institute for Translational Research in Inflammation, Inserm, CHU Lille, University of Lille, 59000 Lille, France; christelneut@nordnet.fr

**Keywords:** endodontic infections, calcium hydroxide, chlorhexidine, copper, silver, zinc, nanoparticle, antimicrobial properties, mechanical properties, rheological properties

## Abstract

The aim of our study was to explore the potential value of metallic (Ag, Cu, and Zn) salts, polymer/metallic nanoparticles, and chlorhexidine (CHX) for improving the antimicrobial activity of calcium hydroxide (CH) against *E. faecalis* and *C. albicans*, associated with persistent endodontic infections. A first screening was performed by determining minimum inhibitory/bactericidal concentrations (MIC/MBC). Antimicrobial activity of the CH paste mixed with metallic salts, chitosan or cyclodextrin polymer metallic nanoparticles was compared to the antimicrobial activity of CH paste alone and CH + CHX using a time-kill kinetics assay. The effect of the antimicrobials on the rheological and the key mechanical properties were also examined. Copper and zinc were discarded because of their MIC/MBC values and silver because of its kill time curve profile. Except for a slower setting time after 24 h and a higher weight loss after 1 week of incubation, the mechanical behavior of the CH paste was unaffected by the addition of CHX. Polymeric/metallic nanoparticles failed to potentiate the antimicrobial effect of CH. By contrast, CHX increased this effect and thus could help eradicate *E. faecalis* associated with persistent root canal infections without altering the desired key physical properties of the CH paste.

## 1. Introduction

Apical periodontitis is an inflammatory disorder of peri radicular tissues caused by persistent microbial infection within the root canal system [1]. The effective elimination of these microorganisms and the prevention of recontamination of the root canal system is the basis of root canal therapy [2]. Even when the most stringent procedures of chemomechanical cleaning and shaping are followed, in some cases, apical periodontitis may persist due to the anatomical complexities of the root canal system [2,3,4]. This is why the complementary application of intracanal antimicrobial medications between sessions has been proposed to significantly reduce the bacterial count [5,6,7].

Calcium hydroxide (CH) paste is the most widely used intracanal medication [8,9]. Its antimicrobial effect has been attributed to the release of hydroxyl ions, which produce a highly alkaline environment (pH 12.5) in which most microorganisms cannot survive [10]. However, it has been suggested that the effectiveness of CH paste may be limited, especially in cases of persistent endodontic infections, due to the presence of resistant microbial species [3,10]. *Enterococcus faecalis* (*E. faecalis*), a facultative anaerobic Gram-positive bacterium, and *Candida albicans* (*C. albicans*), the most common yeast in the oral cavity, are the species most frequently associated with root canal treatment failure and refractory apical periodontitis [3]. They have been detected in 22–77% [3,7] to 1–17% [11] of samples taken from infected root canals. They share characteristics such as dentin penetration ability and high pH tolerance, which may explain their resistance to CH-based intracanal medications [3,10,11].

Various antimicrobial substances have been tested as adjuvants for CH paste in an attempt to increase its antimicrobial activity [12]. Chlorhexidine (CHX) is the reference antiseptic molecule in dentistry and displays a broad spectrum of activity against Gram-positive and Gram-negative bacteria as well as fungi [13,14,15]. It is biocompatible and adsorbs to dental tissues, allowing for prolonged, gradual release at therapeutic levels [13,14,15,16]. The benefit of mixing CH with CHX has been extensively studied [13,15,17,18,19]. Although a number of in vitro and clinical studies have reported that CH + CHX is more effective in eliminating bacteria than CH alone [13,15,18,19,20], other studies have failed to replicate this effect [9,21,22,23]. Differences in drug concentrations, solubility related to the form of the vehicle (aqueous, viscous, or oil), and antimicrobial testing methods may have contributed to this discrepancy [11,13,16]. Metallic nanoparticles (MNPs) have also proven to be effective in controlling oral infections when coated on or incorporated into various materials [24,25,26]. They have been proposed as promising alternatives to traditional antimicrobial agents [27,28,29]. Silver (Ag), copper (Cu), and zinc (Zn) MNPs have received the most attention for their antimicrobial properties [24,25,26,27,30].

Polymeric nanoparticles (PNPs) have been recently developed as sophisticated carriers for drug delivery [31,32,33,34]. They are comprised of active pharmaceutical ingredients that are adsorbed to or entrapped in a polymeric structure [34]. The advantages of PNPs as drug carriers include their improved stability and drug release profiles and their biocompatibility, biodegradability, and ease of production [34]. Metallic nanoparticles have been successfully synthesized in aqueous solutions using chitosan (CHT) or cyclodextrin polymers (CDP) as reducing and stabilizing agents [35,36]. CHT is a biocompatible and biodegradable polysaccharide produced by the deacetylation of chitin extracted from crustaceans, yeasts, and algae [31]. CHT is biodegradable, biocompatible, hemostatic, antimicrobial, and analgesic [31]. These properties are related to its cationic character and the size of the polymer chain [37]. Cyclodextrins (CDs) are cyclic oligosaccharides with glucopyranose units joined by α-1, 4 glycosidic bonds. CDs have a truncated cone-shaped 3D structure. They have a hydrophobic cavity that can form inclusion complexes with a large variety of lipophilic molecules and that can work as a drug carrier in sustained release drug delivery systems [38,39,40,41,42,43,44]. Promising results have been reported with CHT/CDP/metallic nanoparticles. We recently developed a wound dressing with a protective cover composed of CHT and CDP for the sustained release of silver [33]. To date, no studies have investigated the addition of CHT or CDP metallic nanoparticles to CH-based intracanal medications in order to enhance the antimicrobial activity of CH against CH-resistant microorganisms.

The present study compared the in vitro antimicrobial activities of CH paste mixed with different metallic salts or polymer/metallic nanoparticles (PMNPs): CHT/CDP metallic nanoparticles (Ag, Cu, or Zn). CH alone was used as a negative control, and CH + CHX was used as a positive control. The aim was to determine the best intracanal medication candidate to help eradicate *E. faecalis* and *C. albicans* from infected root canals. Since ultimate clinical efficacy also depends on the ability of the material to be easily handled and spread into narrow anatomical spaces [45,46], the key mechanical properties and flowability of the best candidate formulations were also investigated.

## 2. Results

### 2.1. Microbiological Tests

#### 2.1.1. Minimum Inhibitory Concentration (MICs) and Minimum Bactericidal Concentration (MBCs)

Table 1 shows the MICs results for all the antimicrobial agents tested on *E. faecalis* and *C. albicans*. The MICs/MBCs of copper (>3200 mg/L) and zinc (≥3200 mg/L) in free form or as PMNPs reached or exceeded 3200 mg/L, the upper limit of the concentration range studied, meaning that the microbial strains studied were not susceptible to these molecules. This is why the copper- and zinc-based formulations were excluded from further experiments. The MICs (25 to 50 mg/L) and MBCs (50 to >100 mg/L) of silver in free form or as PMNPs and the MICs/MBCs of chlorhexidine (2 to 16 mg/L) indicated that these molecules were bactericidal and bacteriostatic at low concentrations and that the microbial strains studied were susceptible to them. Silver and CHX were thus selected for the next experiments.

#### 2.1.2. Time-Kill Kinetics Assay

**Figure 1:**
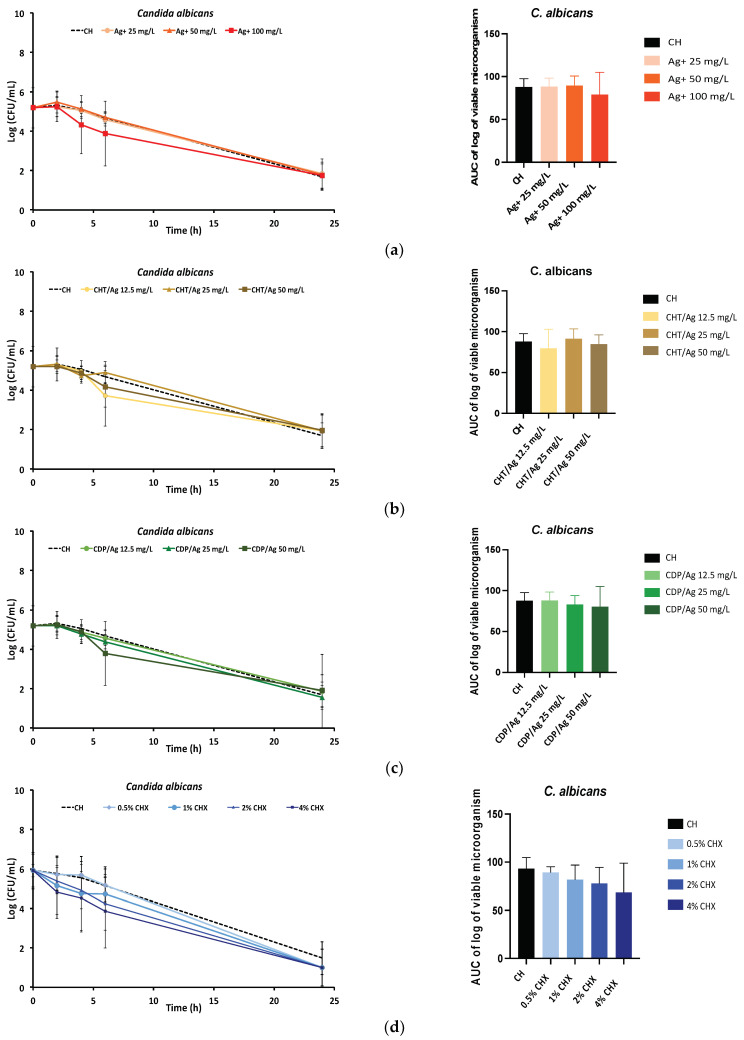

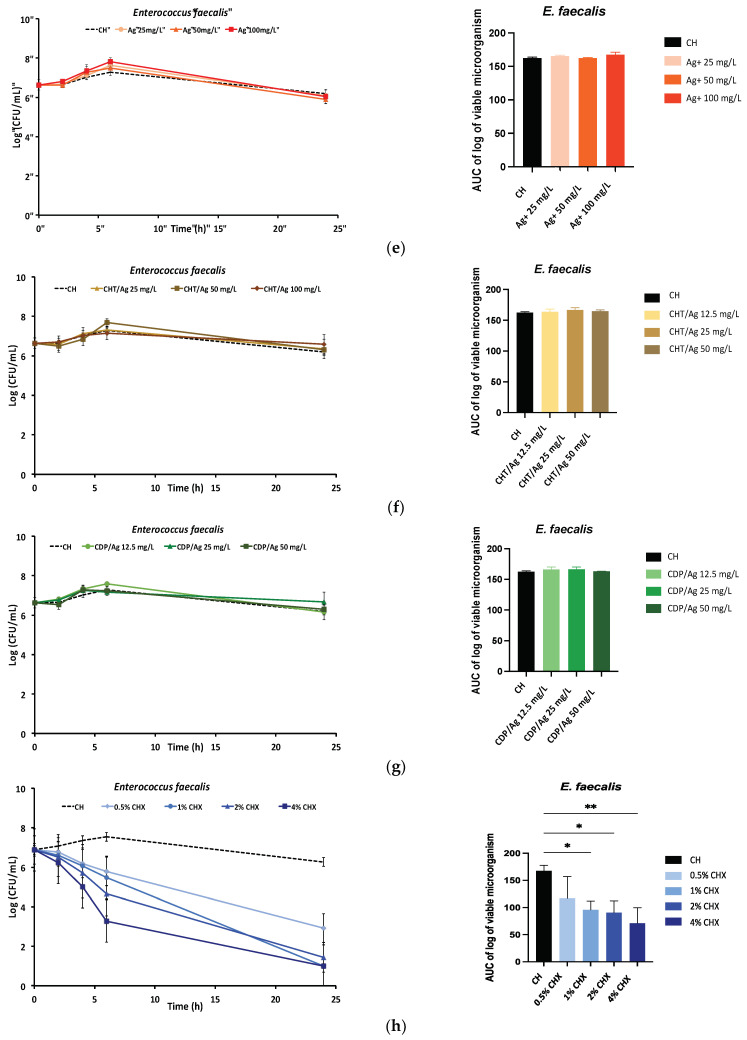
Time-kill kinetics curves and air under the curves (AUC) of the time-kill kinetics for *Candida albicans* (**a**–**d**) and *Enterococcus faecalis* (**e**–**h**) with calcium hydroxide (CH) alone (dotted line) compared to the mixtures (**a**,**e**): CH + silver solution (Ag+) 25 mg/L, 50 mg/L, 100 mg/L; (**b**,**f**) CH + silver as chitosan silver nanoparticles (CHT/Ag) 12.5 mg/L, 25 mg/L, 50 mg/L, 100 mg/L; (**c**,**g**) CH + silver as cyclodextrin polymer silver nanoparticles (CDP/Ag) 12.5 mg/L, 25 mg/L, 50 mg/L, and (**d**,**h**) CH + CHX solution 0.5% (5 × 10^3^ mg/L), 1% (10 × 10^3^ mg/L), 2% (20 × 10^3^ mg/L), 4% (40 × 10^3^ mg/L). (*): significant difference (*p* < 0.05) and (**): significant difference (*p* < 0.01) between the CH formulation and the test formulations (ANOVA test).

The time-kill curve profiles of the CH formulations with silver (Ag^+^, CHT/Ag, CDP/Ag) and CHX against *C. albicans* and *E. faecalis* are presented in Figure 1. Silver was tested at concentrations ranging from 12.5 mg/L to 100 mg/L (Figure 1a–c,e–g), while CHX was tested using four different concentrations ranging from 0.5% (5 × 10^3^ mg/L) to 4% (40 × 10^3^ mg/L) (Figure 1d,h). Similar concentrations have been reported in the literature and correspond to 0.5×, 1×, and 2× MIC for silver and 0.25×, 0.5×, 1×, and 2× the most common concentration used in studies for CHX (2%) [7,24,30,31,47,48]. The time-kill kinetics profile of the drug-free CH paste (dotted line) indicated that there was a reduction in viable *C. albicans* cells but not of *E. faecalis* cells. Silver-loaded CH pastes had similar time-kill kinetics profiles than CH, for all forms (Ag^+^, CHT/Ag, and CDP/Ag) and all concentrations. The time-kill kinetics profile of CHX against *C. albicans* was similar to the control (drug-free CH paste). On the other hand, the time-kill kinetics profile of the CHX-loaded CH paste against *E. faecalis* showed a reduction in the number of viable cells. The antimicrobial effect was dose-dependent. The difference was significant (*p* < 0.05) for the 1% and 2% CHX pastes and was highly significant (*p* < 0.01) for the 4% CHX paste compared to the control (drug-free CH paste). CHX at 1% and 2% displayed the best efficacy at the lowest concentrations against *E. faecalis* and *C. albicans* and were selected for the CHX release tests.

#### 2.1.3. Drug Release Measurements

**Figure 2:**
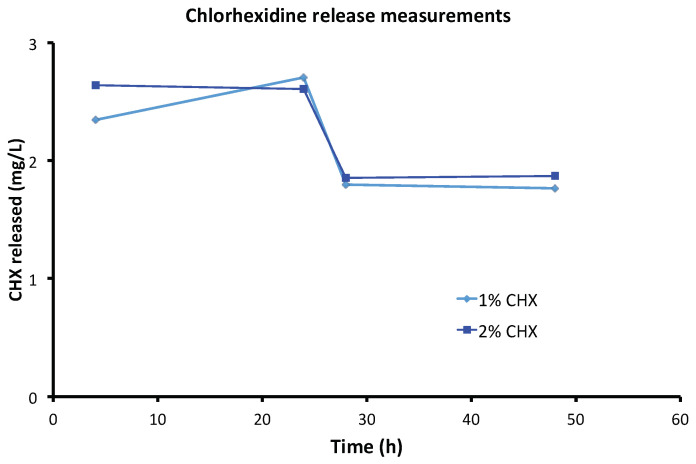
Chlorhexidine release measurements: release of chlorhexidine (CHX) from calcium hydroxide + CHX pastes with 1% (10 × 10^3^ mg/L) and 2% (20 × 10^3^ mg/L) CHX over 48 h.

Chlorhexidine and silver release were determined by HPLC-UV and ICP-MS, respectively, in order to assess the performance of a CH paste as a drug release system. Figure 2 shows the amount of CHX released (mg/L) as a function of time at four time points (4 h, 24 h, 28 h, 48 h) for 1% CHX and 2% CHX. These two dosages displayed comparable antimicrobial activities to 4% CHX (the highest dosage studied) and were thus selected given the risk of dose-dependent CHX toxicity [49]. The 1% CHX/CH paste released 2.350 ± 0.008 mg/L of CHX after 4 h, slightly less than the 2% CHX/CH paste (2.630 ± 0.011 mg/L) (*p* < 0.01), which represent release rates of 0.024% and 0.013% of the initial dosage, respectively. Comparable amounts of CHX were released at 24 h (2.690 ± 0.013 mg/L and 2.600 ± 0.021 mg/L), 28 h (1.790 ± 0.001 mg/L and 1.850 ± 0.009 mg/L), and 48 h (1.760 ± 0.007 mg/L and 1.870 ± 0.005 mg/L) by the 1% CHX/CH and 2% CHX/CH pastes, respectively.

As the time-kill assay indicated that there was no difference in antimicrobial activity between CH alone and Ag-supplemented CH paste, we measured the amounts of silver released in the form of an Ag solution and a PMNP solution to explore a possible correlation between the release profile of the active ingredient and the antimicrobial effect observed. The values for silver after centrifugation as determined by ICP-MS were below the limit of quantification (<0.1 µg/L).

In terms of CHX release, 1% CHX displayed the best efficacy at the lowest concentration and had a better release rate than 2% CHX (0.024% vs. 0.013% at 4 h) and was thus selected for the mechanical tests.

### 2.2. Physical Properties

**Figure 3:**
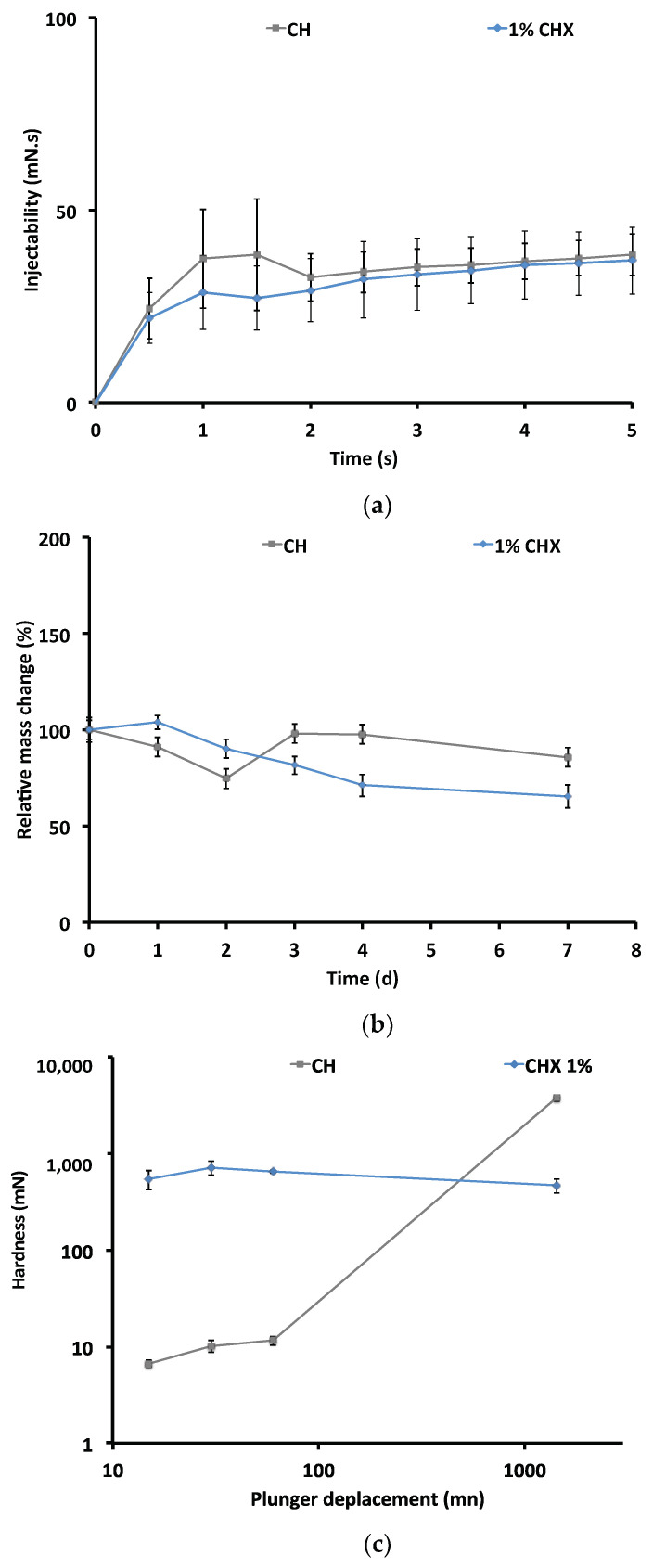
Physical properties of the chlorhexidine-free calcium hydroxide paste (CH) and the calcium hydroxide paste containing 1% chlorhexidine (CHX) (10 × 10^3^ mg/L); (**a**) injectability; (**b**) effect of CHX on mass change; (**c**) setting kinetics.

Figure 3 shows the physical properties of 1% CHX.

#### 2.2.1. Injectability

The results of the mechanical test in terms of the injectability of the CH paste with 1% CHX (10 × 10^3^ mg/L) and the CH paste alone are given in Figure 3a. The two curves have similar profiles, indicating that there was no difference between the two formulations with respect to injectability.

#### 2.2.2. Mass Change

Figure 3b shows the percentage of mass change for the different CH formulations: CHX-free CH paste (CH) and CH + 1% CHX (10 × 10^3^ mg/L) paste. Over a period of seven days, both formulations had mass losses ranging from 15% to 35%. On day 7, the mass loss of the CHX-CH paste was higher (35%) than that of the CHX-free paste (15%).

#### 2.2.3. Puncture Resistance Test

Figure 3c shows the hardening curves. The initial hardness of the CH paste increased with the addition of CHX (292 ± 25.11 mN vs. 5.29 ± 0.23 mN). The hardness of the CHX-free CH paste increased over time. At the end of the test, the hardness of the CHX-free formulation (3767.74 ± 265.86 mN) was higher than that of the CHX- supplemented formulation (468.33 ± 78.42 mN).

### 2.3. Rheological Properties

**Figure 4:**
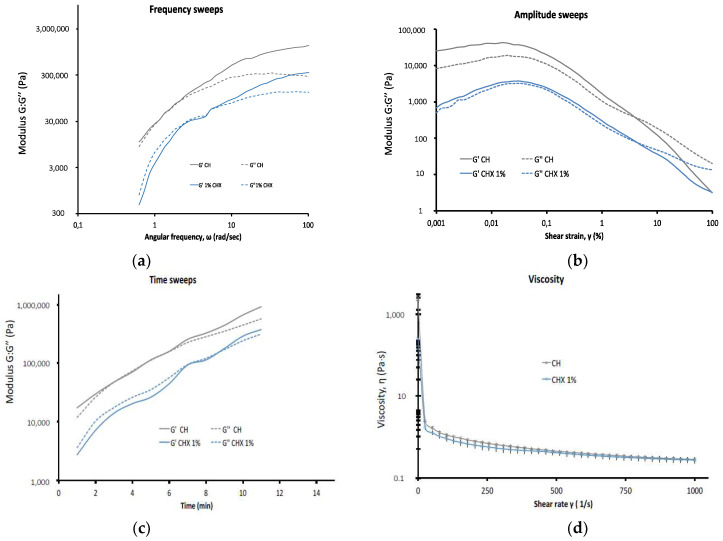
The rheological properties of the calcium hydroxide paste formulations: chlorhexidine-free calcium hydroxide paste (CH) and calcium hydroxide with 1% chlorhexidine (CHX) (10 × 10^3^ mg/L): (**a**) frequency sweeps: determining G′ and G″ at a constant strain of 0.01%; (**b**) amplitude sweeps: determining G′ and G″ at a different shear strain; (**c**) time sweeps: evolution of the storage (G′) and loss (G″) moduli as a function of time at a frequency of 2.5 rad/s at 20 °C; (**d**) viscosity: shear-thinning evaluation by shear-rate dependent variations in viscosity.

The rheological properties of the calcium hydroxide formulations are shown in Figure 4.

The frequency sweep tests (Figure 4a) and the amplitude sweep tests (Figure 4b) were performed to determine the rheometric parameters. One preview test was performed for each sealer to analyze the setting pattern and to determine the rheometer settings (angular frequency of 2.5 rad/s) using a strain amplitude of 0.01% and the data analysis method.

For both formulations, G’ and G″ increased over time. On the time sweep analysis curves (Figure 4c), the storage moduli (G’) were very close to the loss moduli (G″) (G’ = G″ approximately) for both formulations. For the CHX-free formulation, after 6 min, G’ became higher than G”. In the presence of 1% CHX, the two moduli (G’ and G″) distanced themselves from each other: G″ was first higher than G’ then, after 9 min, G″ became lower than G’.

The amplitude sweeps showed that the moduli were lower with 1% CHX than without CHX. For both formulations, the flow point was 0.06%. The G points were 3.93% for the CHX-free formulation and 4.95% for the 1% CHX formulation. Figure 4d shows the formulation viscosity during the first 15 min. Both formulations displayed the same behavior but the CHX-free formulation was mechanically more consistent than the CHX formulation. Indeed, the initial viscosity was 2320 Pa·s for the CH paste alone and 253 Pa·s with the CH + CHX paste, following which the values became similar for both formulations.

## 3. Discussion

The present study compared the in vitro antimicrobial activity of a CH paste mixed with different metallic salts (Ag_2_SO_4_, CuSO_4_, ZnCl_2_) or CHT/CDP metallic nanoparticles to CH alone as a negative control and the CH + CHX paste as a positive control. The aim was to explore the potential value of metallic salts and CHT and CDP metallic nanoparticles as antimicrobial adjuvants for CH paste for eradicating *E. faecalis* and *C. albicans*, which are associated with root canal infections and are resistant to CH [3,10,11]. According to our results, only CHX was an effective antimicrobial adjuvant for CH paste, reducing the number of *E. faecalis* colonies after 24-h incubation.

The dosages of CHX incorporated into the CH paste were selected according to the most commonly used concentrations in similar studies [13,14,15,18,19,20,22,25] for comparison purposes. For the metallic salts, in particular the PMNPs, which are still very little studied as intracanal medications, the antimicrobial susceptibilities of *E. faecalis* and *C. albicans* were first verified using a standard MIC/MBC determination method (CLSI M07-A8), and the dosages to be tested were selected based on the MICs (0.5×, 1×, and 2×), as previously described [50,51,52]. At this first screening stage, copper and zinc (whose MIC values reached or exceeded 3200 mg/L for both *E. faecalis* and *C. albicans*) were excluded. Conflicting results have been reported in the literature regarding the susceptibility of *C. albicans* and *E. faecalis* to zinc and copper. Aghatabay et al. and Martínez et al. reported MIC values for *C. albicans* reaching 12.5 mg/L for zinc and 129.7 mg/L for copper, respectively [53,54]. More recently, Meto et al. reported a similar *C. albicans* MIC for copper mixed with CH (125 mg/L) [55]. Nozari et al. reported that they did not observe any antimicrobial effect by zinc oxide on *E. faecalis* [56]. Conflicting results have also been reported with the same metals for other oral bacteria, including *Streptococcus mutans* [57]. It has been suggested that some metals may be inactivated by complex bacterial culture media [58] or that the discrepancies may be due to the different strains of the bacteria used in each study [54,55]. The MICs observed for silver salts and nanoparticles in the present study are generally consistent with those reported in previous studies [59,60,61].

As the results of the preliminary antimicrobial susceptibility test clearly indicated that the microorganisms studied are not susceptible to copper and zinc, only chlorhexidine and silver were selected to investigate their potential for enhancing the antimicrobial activity of the CH paste. A time-kill kinetics assay was used to monitor bacterial growth and death over a wide range of antimicrobial concentrations over time [62]. We used this technique rather than agar diffusion, which is the most commonly used technique to assess the antimicrobial activity of intracanal medications [63], given that the agar diffusion test has repeatedly been shown to be unreliable and unreproducible due to a number of limitations [64]. These limitations include reactions between agar ingredients and the antimicrobial agent, the lack of differentiation between bactericidal and bacteriostatic activity, variations related to the thickness of the agar gel, and the choice of cut-off size for the inhibition zones [64,65,66].

Three main conclusions can be drawn from our results with respect to the potential interest of antimicrobial-supplemented CH paste. First, our results suggested that the addition of silver in the form of a sulfate salt (Ag_2_SO_4_), CHT/Ag, or CDP/Ag does not improve the antimicrobial activity of CH against *E faecalis.* A recent study has shown that the antibacterial efficacy of a silver nanoparticle (AgNP) + CH combination is superior to CH alone when using single bacteria species or biofilms [67] while another study failed to show this benefit [24]. These discrepancies are generally attributed to differences in methodology or to the characteristics of the nanoparticles used. Pratsinis et al. have shown that the antibacterial activity of AgNPs as well as their toxicity are size-dependent [68]. AgNPs that are less than 10 nm in size have enhanced antimicrobial activity and are more toxic. This has been attributed to a larger available surface area for interactions with microorganisms and a greater capacity to penetrate the bacteria [61,68,69,70,71,72]. Increasing evidence has also shown that AgNP activity may also depend on shape. It has been reported that triangular nanoparticles may be more effective than spherical and rod-like shapes. It has been suggested that a triangular shape gives a greater positive charge to the nanoparticles, which may, in turn, contribute to better antimicrobial activity [61]. In the present study, the fact that the CHT/Ag- and CDP/Ag-containing CH pastes had no antibacterial activity is most likely due to a lack of silver ion release into the medium. Indeed, the ICP-MS analysis showed that no Ag was detected in the bacterial culture medium after 24 h (<0.1 µg/L). Polymer/silver or polymer/CH interactions may explain this observation, but given the lack of studies on CH + PMNP mixtures as intracanal medications, further investigations are needed to elucidate this issue.

Second, our results indicated that the CH + CHX combination is more effective in reducing the bacterial count of *E. faecalis* than CH paste alone and that this effect is dose-dependent, which is consistent with the known properties of CHX as an antibacterial agent [13]. A large number of recent studies have reported similar results [13,15,18,19,20,30], but the benefit of mixing CH with CHX to improve the antibacterial activity of CH remains controversial. A systematic review and meta-analysis by Saatchi et al., which included nine studies, failed to show a significant difference between CH + CHX and CH alone in their effects on *E. faecalis* [63]. However, it should be mentioned that the most recent studies (published after 2012) were not included in this systematic review and that the comparisons made in the meta-analysis were very heterogeneous.

Lastly, we observed the same patterns of reduction of microbial counts between 1 h and 24 h of incubation of *C. albicans* in the presence of CH alone or in combination with the different antimicrobials. This means that the CH paste alone or combined with any of the antimicrobials studied has comparable fungicidal activity against *C. albicans*. This result contradicts the resistance of *C. albicans* to CH that is commonly documented in the literature [3,55,73]. Waltimo et al. studied the susceptibility of common oral Candida species to a saturated aqueous CH solution and reported that the sensitivity to CH was variable. Only 6 of 16 strains survived after 3 h but were killed after 6 h of incubation [73]. Similar results were reported by Weckwerth et al., who studied the susceptibility of 30 strains of oral *C. albicans* isolated from clinical specimens [74]. A decline in *Candida* growth was observed at 6, 12, and 24 h, with full inhibition of growth after 48 h of direct contact with an aqueous CH solution [74]. However, it seems evident that the resistance of *C. albicans* to CH in vivo is higher because the microorganisms are able to invade dentinal tubules and remain protected from the action of alkaline endodontic medications because of the buffering effect of dentin [66,75,76].

The key characteristics of an ideal endodontic filling material remain as stated by Grossman in [77]. In particular, from a physical and mechanical point of view, the filling material is expected to (i) be easy to handle, (ii) have an ample working time, (iii) be adaptable to the complex internal anatomy of root canals, and (iv) be dimensionally stable and insoluble in tissue fluid. It should be noted that this last point does not apply in the same way to temporary filling materials such as CH-based medications, which must be completely removed from the canal before the final obturation as the presence of CH paste remnants may negatively affect the quality of the root canal filling and reduce dentin bond strength and sealer adaptation [78]. The flowability of the filling material is clinically important because it improves the penetration of the sealer into the complex root canal, thus contributing to the clinical performance of the material [79,80,81]. This parameter can be precisely studied using a strain-controlled rheometer that provides information on the rheological properties of root canal sealers as a function of time and temperature [79]. Relatively few studies have investigated the rheological properties of root canal sealers [82,83] and, to our knowledge, none have investigated CH-based temporary medications. Injectability and penetration resistance tests are also standard methods for evaluating the ease of handling of injectable materials and the setting time [62,84]. Monitoring mass changes in water also provides information on water uptake/loss, which contributes to the swelling and/or disintegration of the material [62,84]. This is why we used the above methods to characterize the key physical properties of the CH + 1% CHX mixture. This formulation displayed the best efficacy at the lowest drug concentration. A slight rheofluidifying effect of CH + CHX (G” > G’) at 9 min vs. 6 min for CH alone was observed but, overall, the rheological properties and ease of handling of the CH paste were not altered by the addition of CHX, as shown by the similar viscosity and injectability curves for the two formulations. Moreover, the addition of 1% CHX seemed to slow down the setting of the CH paste and promote mass loss over time. The CH + CHX mixture had lower penetration resistance at 24 h and a higher mass loss after 7 days. The hydrophilic nature of CHX may explain these differences as it would promote the hydration of the CH paste, which would delay its hardening. In practice, the faster mass loss of the CH + CHX mixture could reflect faster resorption of the material in vivo due to its solubilization by tissue fluids.

The limitations of the present study are evidently related to the in vitro models used, which only made it possible to approximate a few of the parameters that could be involved in vivo. Notably, antimicrobial activity was evaluated with planktonic bacteria. In vivo, endodontic pathogens are organized in the form of polymicrobial biofilms whose susceptibility to antimicrobial agents can be very different. Further studies are required to confirm these results and correlate them with clinical outcomes.

## 4. Materials and Methods

### 4.1. Media, Molecules, and Formulations

#### 4.1.1. Cultures and Media

Different types of tests, which are described in detail below, were performed using either a collection strain (*C. albicans* ATCC10231) or a strain isolated from a patient with a periapical lesion (*E. faecalis* C159.6). These strains were obtained from the Laboratory of Bacteriology collection at the College of Pharmacy, University of Lille, France. The two strains were subcultured in Mueller-Hinton broth (MHB composed of MH culture medium and ultra-pure water), seeded on Mueller Hinton agar (MHA composed of Mueller-Hinton culture medium (Oxoid^®^, Basingstoke, UK) and 1.5% agar (Becton-Dickinson^®^, Le Pont de Claix, France)) diluted in cysteinated Ringer (RC) solution (Merck^®^, Darmstadt, Germany).

#### 4.1.2. Preparation of Antimicrobial Agents

The antimicrobials were tested in the form of solutions: chlorhexidine digluconate (Evonik, Hanau, Germany), silver sulfate (Ag_2_SO_4_) (Sigma-Aldrich^®^, Saint-Quentin Fallavier, France), copper (II) sulfate anhydrous (CuSO_4_) (Alfa Aesar^®^, Kandel, Germany), and zinc chloride anhydrous (ZnCl_2_) (Alfa Aesar^®^). Metallic ions (silver (Ag), copper (Cu), and zinc (Zn) were also tested in the form of PMNPs and were formulated with (i) low molecular weight chitosan (CHT) (MW: 140 kg mol^−1^; DD: 77%) (Sigma-Aldrich^®^) to produce chitosan/metallic nanoparticles (CHT/MNPs) and were named, respectively, CHT/Ag, CHT/Cu, and CHT/Zn or (ii) with cyclodextrin polymer (Kleptose^®^, Roquette, Lestrem, France) to produce cyclodextrin polymer/metallic nanoparticles (CDP/MNPs) named, respectively, CDP/Ag, CDP/Cu, and CDP/Zn, as previously described [33,36].

An aqueous CHX solution was prepared by diluting 1024 mg of lyophilized digluconate CHX powder in 100 mL of distilled water. The metallic solutions (Ag^+^, Cu^2+^, Zn^2+^) were prepared by diluting 640 mg of Ag_2_SO_4_, CuSO_4_, or ZnCl_2_ in 100 mL of ultra-pure water.

The aqueous CHT/Ag, CHT/Cu, CHT/Zn nanoparticle solutions (0.5% chitosan) were prepared in an acid medium (1% *v*/*v* lactic acid). The Ag_2_SO_4_, CuSO_4_, and ZnCl_2_ solutions (6400 mg/L) were prepared in volumetric flasks containing 100 mL of a 1% (*v*/*v*) aqueous lactic acid (VWR^®^, Fontenay Sous Bois, France) solution in ultra-pure water. The appropriate amount (500 mg) of chitosan was then mixed with the lactic acid solution and was stirred for 24 h. Once the chitosan solution became transparent, 640 mg of Ag_2_SO_4_, CuSO_4_, or ZnCl_2_ was added to the volumetric flask to obtain 100 mL of each CHT/MNP solution. A similar procedure was used for the production of aqueous CDP/Ag, CDP/Cu, and CDP/Zn nanoparticle solutions containing 0.3% cyclodextrin polymer with 6400 mg/L of Ag_2_SO_4_, CuSO_4_, or ZnCl_2_. These solutions were prepared in 100-mL volumetric flasks. The appropriate amount (300 mg) of cyclodextrin polymer was mixed in ultra-pure water and was stirred overnight (50 rpm). Next, 640 mg of Ag_2_SO_4_, CuSO_4_, or ZnCl_2_ was added to the volumetric flasks to produce 100-mL solutions of CDP/MNPs. The CHT/MNP and CDP/MNP solutions were stirred for 24 h at 70 °C in the dark.

#### 4.1.3. Formulation of the Calcium Hydroxide Pastes

CH paste was prepared by mixing calcium hydroxide powder (Hidroxido de calcio; DentaFlux, Madrid, Spain) and distilled water (1:1; *w*/*v*) and spatulating the mixture on a glass plate. Antimicrobial-free CH paste was used as a negative control. The test formulations were produced by replacing the distilled water with the antimicrobial solutions described above (CHX, Ag^+^, Cu^2+^, Zn^2+^, CHT/Ag, CHT/Cu, CHT/Zn, CDP/Ag CDP/Cu, or CDP/Zn).

### 4.2. Antimicrobial Properties

#### 4.2.1. Minimal Inhibitory Concentrations and Minimal Bactericidal Concentrations

The MICs and MBCs were determined using Clinical and Laboratory Standards Institute protocols (CLSI M07-A9, CLSI M26-A) [85]. Briefly, the broth microdilution method was used with ten concentrations of the antimicrobials (chlorhexidine digluconate solution, and silver, copper, and zinc solutions or in the form of PMNPs) [86]. The MICs and MBCs of *C. albicans* and *E. faecalis* were determined in triplicate, with antimicrobial concentrations ranging from 1 to 3200 mg/L, depending on the antimicrobial agent. A start inoculum of 10^6^ cells/mL was used. The MICs were the lowest concentrations of the antimicrobials with which no visible growth occurred. The CFUs were visible after a 24- to 48-h incubation at 37 °C under aerobic conditions. The MBCs were the lowest concentrations at which no colony formation occurred. The first selection of antimicrobials was made based on the MIC and MBC results. The antimicrobials with the lowest MICs and MBCs were selected for the next step.

#### 4.2.2. Time-Kill Kinetics Assay

A time-kill kinetics assay was performed to determine the time-dependent reduction in *E. faecalis* and *C. albicans* (Colony Forming Units [CFUs]) caused by the antimicrobials, as previously reported [62]. Briefly, bacterial suspensions were exposed to the antimicrobials for different periods of time, and the CFUs of the surviving microbial populations were counted. Each formulation (1 mL) was placed in a 15-mL Falcon tube (Greiner, Courtaboeuf, France). Fresh MH medium was added (8 mL) followed by 1 mL of *E. faecalis* or *C. albicans* suspension (10^4^–10^6^ bacteria/mL). The tubes were then incubated for 24 h at 37 °C. At 0, 2, 4, 6 and 24 h, 100-µL samples were removed from the tubes and were diluted 10-fold with RC. The dilutions (100 µL) were plated on MHA plates, which were incubated for 24 h at 37 °C. The numbers of colonies were counted, and the results are expressed as log CFU/mL. All experiments were performed in triplicate, and the results are expressed as means ± standard deviations.

#### 4.2.3. Drug Release Measurements

The antimicrobials (100 µL) were placed in the bottom of 2-mL Eppendorf^®^ vials using a standard syringe. The vials were horizontally shaken at 37 °C and 80 rpm (GFL 3033; Gesellschaft fuer Labortechnik, Burgwedel, Germany). At selected time points, the release medium (deionized water) was completely renewed. The amounts of drugs in the bulk fluids that were withdrawn from the vials were determined by HPLC-UV (Waters Alliance 2695 separation module, Waters 2489 UV/vis detector), as previously described [87]. Briefly, 20-μL samples were injected onto a C18 RP column (Gemini 5 μm C18 110 Å, 100 mm Å~4.6 mm; Phenomenex, Le Pecq, France). The flow rate was 1.5 mL/min. The column was kept at room temperature, and the detection wavelength was set at λ = 239 nm. All experiments were performed in triplicate, and the results are expressed as means ± standard deviations.

### 4.3. Physical Properties

Depending on the results of the antimicrobial test, the antimicrobial agents displaying the best antibacterial properties were tested mechanically.

#### 4.3.1. Injectability

The injectability of the different CH paste formulations was measured using a texture analyzer in compression mode (speed: 1 mm/s), as previously described [88]. The work required to expel the CH paste was calculated. Briefly, a cylindrical probe (6 mm in diameter) of the texture analyzer was used to drive the piston 10-mm downward (load cell: 50 kg, TA.XT.Plus; Stable Micro Systems, Surrey, UK). Force-distance profiles were recorded, and the work required to expel the liquid formulation from the syringe was calculated. For comparison purposes, the injectability of pure water was also determined. All the experiments were performed in triplicate, and the results are expressed as means ± standard deviations.

#### 4.3.2. Monitoring Changes in Dynamic Mass

The mass change % at time *t* was calculated, as previously described [87], using the following equation:

$$\mathrm{mass}\;\mathrm{change}\;\left(\%\right)\left(t\right)=100\cdot \frac{\mathrm{mass}\;\left(t\right)-\mathrm{mass}\left(t=0\right)}{\mathrm{mass}\;\left(t=0\right)},$$

where the mass (*t* = 0) is the initial weight of the CH paste formulation used. The experiments were performed in triplicate, and the results are expressed as means ± standard deviations.

#### 4.3.3. Penetration Resistance Test

The setting profiles of the CH paste samples were determined by measuring their penetration resistance at different times (15 min, 30 min, 60 min, 24 h) using an adaptation of a previously published protocol [84]. Briefly, a cylindrical hole was cut in the center of an agar gel and was filled with 200 μL of CH paste. A texture analyzer (TA.XT.Plus, load125cell: 1 kg) in compression mode and a spherical probe (6 mm) were used for the puncture test. Once in contact with the CH paste sample, the probe was lowered downward at a constant speed (1 mm/s) and was stopped when its penetration depth was equal to the target distance (5 mm). The force-displacement profile was recorded, and the area under the force-time plot was calculated. The tests were performed in triplicate at room temperature.

### 4.4. Rheological Properties

The rheological properties were evaluated using a strain-controlled modular compact rheometer (MCR 301 Physica: Anton Paar, Les Ulis, France) with a parallel plate geometry (diameter of 25 mm) and a Peltier plate temperature device (HPTD200, Anton Paar) to control the temperature [89]. A 1-mm gap was used for all the measurements. The CH paste samples were placed on the lower plate of the rheometer immediately after mixing. The upper plate was placed in the measuring position, and excess sample was trimmed using a spatula. The storage (G′) and loss (G′′) moduli of the paste samples were evaluated as a function of time at 20 °C in the oscillatory mode at a frequency of 2.5 rad/s, a 0.01% strain, and a constant speed of 300 s^−1^. The measurements began 90 s after the initial mixing in order to standardize the measurements. The tests were performed in triplicate for each group.

### 4.5. Statistical Analysis

The values were tabulated using Microsoft Office Excel Mac OS 2011 (14.4.7 [141117] version). The significance level of the statistical analysis was set at *p* < 0.05. The time-kill assay test results were analyzed by ANOVA followed by a Tukey Kramer test. All the experiments were repeated independently at least three times.

## 5. Conclusions

Within the limits of the present study, we can conclude that a mixture of CH and CHX as an intracanal medication could help eradicate *E. faecalis* associated with secondary and persistent root canal infections without altering the desired key physical properties of the CH paste. Our results do not support the use of CHT or CDP metallic nanoparticles as antimicrobial adjuvants for CH-based medication. Further studies are needed to optimize the antimicrobial activity of CHT/CDP metallic nanoparticles before they can be used as an adjuvant for endodontic therapy.

## Figures and Tables

**Table 1:** Minimal inhibitory concentrations (MICs) and minimal bactericidal concentrations (MBCs) (mg/L) of copper (Cu^2+^), zinc (Zn^2+^), silver (Ag^+^), and chlorhexidine (CHX) solutions, chitosan metallic nanoparticles (CHT/Cu, CHT/Zn, CHT/Ag), and cyclodextrin polymer metallic nanoparticles (CDP/Cu, CDP/Zn, CDP/Ag) against *Enterococcus faecalis* and *Candida albicans* (*n* = 3).

Antimicrobials Agents	*Enterococcus faecalis* MIC/MBC (mg/L)	*Candida albicans* MIC/MBC (mg/L)
Cu^2+^	>3200	>3200
CHT/Cu	>3200	>3200
CDP/Cu	>3200	>3200
Zn^2+^	3200	3200
CHT/Zn	3200/>3200	3200/>3200
CDP/Zn	3200	3200
Ag^+^	50/>200	50
CHT/Ag	50/>100	25/50
CDP/Ag	25/>100	25/50
CHX	2/16	4

## Data Availability

The data presented in this study are available on request from the corresponding author.
